# Factors affecting statin uptake among people living with HIV: primary care provider perspectives

**DOI:** 10.1186/s12875-021-01563-0

**Published:** 2021-10-30

**Authors:** Allison J. Ober, Sae Takada, Deborah Zajdman, Ivy Todd, Tamara Horwich, Abraelle Anderson, Soma Wali, Joseph A. Ladapo

**Affiliations:** 1 RAND Corporation, Santa Monica, CA USA; 2 Division of General Internal Medicine and Health Services Research, Department of Medicine, UCLA Geffen School of Medicine, Los Angeles, CA USA; 3 Division of Cardiology, Department of Medicine, UCLA Geffen School of Medicine, Los Angeles, CA USA; 4 Olive View – UCLA Medical Center, Sylmar, CA USA

**Keywords:** Cardiovascular disease, ASCVD risk assessment, Statin therapy, people living with HIV

## Abstract

**Background:**

Cardiovascular disease (CVD) is a major cause of morbidity and mortality among people living with HIV (PLWH), but statin therapy, safe and effective for PLWH, is under-prescribed. This study examined clinic leadership and provider perceptions of factors associated with statin prescribing for PLWH receiving care in eight community health clinics across Los Angeles, California.

**Methods:**

We conducted semi-structured telephone interviews with clinic leadership and providers across community health clinics participating in a larger study (INSPIRE) aimed at improving statin prescribing through education and feedback. Clinics included federally qualified health centers (*N* = 5), community clinics (*N* = 1) and county-run ambulatory care clinics (*N* = 2). Leadership and providers enrolled in INSPIRE (*N* = 39) were invited to participate in an interview. We used the Consolidated Framework for Implementation Research (CFIR) to structure our interview guide and analysis. We used standard qualitative content analysis methods to identify themes within CFIR categories; we also assessed current CVD risk assessment and statin-prescribing practices.

**Results:**

Participants were clinic leaders (*n* = 6), primary care physicians with and without an HIV specialization (*N* = 6, N = 6, respectively), infectious diseases specialists (*N* = 12), nurse practitioners, physician assistants and registered nurses (*N* = 7). Ninety-five percent of providers from INSPIRE participated in an interview. We found that CVD risk assessment for PLWH is standard practice but that there is variation in risk assessment practices and that providers are unsure whether or how to adjust the risk threshold to account for HIV. Time, clinic and patient priorities impede ability to conduct CVD risk assessment with PLWH.

**Conclusions:**

Providers desire more data and standard practice guidance on prescribing statins for PLWH, including estimates of the effect of HIV on CVD, how to adjust the CVD risk threshold to account for HIV, which statins are best for people on antiretroviral therapy and on shared decision-making around prescribing statins to PLWH. While CVD risk assessment and statin prescribing fits within the mission and workflow of primary care, clinics may need to emphasize CVD risk assessment and statins as priorities in order to improve uptake.

## Background

As life expectancy of people living with HIV (PLWH) approaches that of HIV-uninfected adults, [1, 2] prevention of cardiovascular disease (CVD) has become critical for decreasing morbidity and mortality among PLWH [3–6]. PLWH have a higher prevalence and earlier onset of CVD. However, evidence-based statin therapy—which is safe (e.g., pitavastatin/atorvastatin) and highly effective at reducing cardiovascular risk [7, 8] and all-cause mortality [9] among PLWH —is under-prescribed for PLWH [10, 11]. Although there remains some debate over the independent effect of LDL-C on cardiovascular risk, statins have been robustly demonstrated to reduce cardiovascular risk, and PLWH are less likely to be prescribed statins compared to HIV-uninfected adults [12]. Of note, the QRISK3 found that HIV was associated with a 25% increased risk in women and a 17% increased risk in men. While these risk estimates did not meet statistical significance at the 0.01 level potentially due to a relatively low event rate in PLWH attributable to the cohort’s younger age, [13] multiple modeling and epidemiologic studies overall have found that increased cardiovascular risk among patients with HIV [3–6].

Barriers to statin use for PLWH have not been well-studied but could be similar to barriers documented for other highly effective but underused therapies. For example, in prior research on impediments to prescribing treatment for hepatitis C among PLWH, barriers among providers include low self-efficacy, concerns about side effects, and knowledge gaps about guidelines, as well as concerns about patient adherence, particularly for PLWH who already have difficulty adhering to their antiretroviral treatment regimen [14, 15]. Provider-level barriers common to the adoption of new practices more broadly include perceived complexity and effectiveness of the practice, fit with and relevance to existing practices, and feasibility of implementing the practice [16]. Primary care providers may also face additional barriers such as large workloads, imbalance between expertise and increasing job demands, and lack of team support [17–19]. At the patient level, barriers to statin uptake in the general population include medication burden, concerns about side effects, and uncertainty about benefits [20–23]. For the care of PLWH specifically, barriers to statin uptake could include those that interfere with antiretroviral therapy and implementation of primary care guidelines more generally, such as lack of perceived need for treatment, lack of engagement in medical care, and psychosocial factors and life circumstances such as mental health issues, drug and alcohol use, stigma, and unstable housing [14, 24–26].

Consistent with this literature, implementation science research suggests that factors affecting implementation of evidence-based practices occur across multiple levels of a health system, and that barriers and facilitators at each level must be understood and addressed for successful implementation to occur [27]. This study aimed to assess through qualitative interviews with primary care clinic leadership and providers clinic- provider- and patient-level factors affecting statin uptake in community-based primary care clinics, where PLWH are most likely to receive health care. This article describes findings from these interviews.

This study is part of the INSPIRE study, a stepped-wedge cluster randomized trial to test the effects of a multi-level implementation strategy with two interventions, education and peer comparison feedback, on rates of statin prescribing to PLWH by their primary care providers [28].

## Methods

### Study setting

Data was collected from clinic leadership and providers participating in the INSPIRE study. (This article refers to all physicians, NPs, PAs, and RNs who participated in the study as “providers,” but differentiates between provider types where relevant differences emerged.) The study is taking place in eight public community health clinics in Los Angeles County, California that serve PLWH. Clinics fall into four categories: Federally qualified health centers (FQHC) (*N* = 5); non-FQHC community clinics (*N* = 1), county-based ambulatory care clinics (CACC) (N = 1), and CACC within a hospital setting (N = 1). All clinics serve a racially/ethnically diverse population of PLWH, focus on the care of underserved populations, and include a mix of providers providing primary care services including infectious disease specialists, primary care physicians with and without an HIV specialization, nurse practitioners and physician assistants, and registered nurses.

This study was approved by the University of California, Los Angeles Institutional Review Board. All participants provided informed signed consent to participate in the larger study (INSPIRE). Prior to the interviews, participants were reminded that they had provided informed consent for the larger study and then were asked to provide informed verbal consent to participate in the interview and to be recorded. All participants provided informed consent prior to participation.

All methods were carried out in accordance with relevant guidelines and regulations.

### Procedures

We conducted semi-structured telephone interviews with leadership and providers across participating clinics in the INSPIRE study. Thirty-nine clinic leaders and providers enrolled in INSPIRE were invited toparticipate in an interview between April 2019 and April 2020. Following a study kickoff meeting for INSPIRE, we contacted consented providers by email and followed up by telephone to schedule an interview. Interviews lasted up to 45 min. Participants were offered a $125 gift card for completing the interview, which was mailed to them if they agreed to receive it. Interviews were recorded and transcribed. Participants were reminded of study procedures and provided their informed verbal consent to participate and be recorded.

### Measures

We developed an interview guide based on the Consolidated Framework for Implementation Research (CFIR) [29]. The CFIR is a “meta-theoretical” framework that draws upon organizational and behavioral change theories of factors critical to implementation of evidence-based practices and consolidates them into a single framework that offers a menu of constructs. We based the guide on the following CFIR domains thought to influence uptake of evidence-based practices: *Characteristics of Statins* (based on CFIR category “Characteristics of the Innovation”), pertaining to statin effectiveness, ease of use, relative advantage over other interventions (such as lifestyle changes); *Characteristics of Providers* (based on the CFIR category “Characteristics of Individuals”), inclusive of themes related to provider knowledge or knowledge gaps, self-efficacy and training; *Inner Setting*, encompassing structural or clinic-related themes including leadership support and fit of statin prescription with current practices, workflows and clinic mission and priorities; and *Patient Factors* (in CFIR included in the category “Outer Setting”), incorporating themes related to patient characteristics, needs and resources. We added a category called *Current Practices* to capture practices currently used to assess CVD risk and prescribe statins. Initial questions were broad and open-ended and were followed up with closed-ended questions to clarify responses and obtain greater detail. Leadership and provider interview guides varied slightly, e.g., leadership interviews asked about barriers to provider statin prescribing generally, not necessarily referring to their own prescribing practices. Interview guides are available upon request.

### Analysis

The research team (investigator AO; research assistants (RAs) IT, DZ) read all transcripts and, using the codebook, came to consensus on subdomains of emergent themes. Using Dedoose (a qualitative analysis software program), the team first entered CFIR domains and subdomain themes into the codebook. IT and DZ then marked areas of text pertaining to each domain and construct code. IT and DZ practiced with a random sample of 20% of transcript sections, coding independently and reviewing together. If coder disagreement revealed ambiguity in the codebook, we modified the codebook. Training continued until the two coders could consistently identify and mark each theme. Next, both coders each worked on two interviews independently, after which measured coder consistency was assessed. Once consistency was reached, evidenced by Kappas of ≥0.70 considered “good” consistency [30], they coded the remainder of the transcripts independently. Themes that did not fall into one of the CFIR domains were marked as “other.” The analysis team categorized these themes and added them to the codebook. The RAs then marked text pertaining to these codes. Differences between provider and clinic types were examined.

## Results

Thirty-seven of the 39 participants from the larger study participated in an interview. Sixteen percent were clinic leaders (CL). (To protect CL identities, we do not indicate clinic type of CL in quote attributions.) About one third (32%) of participants were infectious disease (ID) specialists. Sixteen percent each were primary care physicians without specialization in ID (PCP) and primary care physicians who had completed an HIV specialization (PCP-HIV). Nineteen percent were nurse practitioners (NP), physician assistants (PA), or registered nurses (RN). (Despite differences in background and training, these provider types are combined into a single category to protect identification of the small numbers of providers in some provider categories*.*). Most of the ID were in a hospital-based CACC, whereas the majority of PCP or PCP-HIV were in an FQHC or non-FQHC community clinic. (See Table 1.)

**Table 1:** Participants

Participant Type	All		Clinic Type							
			Federally Qualified Health Center (FQHC)		Non-FQHC Community Clinic (CC)		County Ambulatory Care Clinic (CACC)		Hospital-based CACC (CACC-Hospital)	
	**N = 37**		***N*** **= 19**		***N*** **= 4**		**N = 4**		***N*** **= 10**	
	N	%	N	%	N	%	N	%	N	%
Clinic Leadership (Chief Medical Officer, Executive Director, Program Manager) (CL)	6	14%	4	21%	1	25%	1	25%	0	0%
Infectious Disease Specialist (ID)	12	35%	3	16%	0	0%	1	25%	8	80%
Primary Care Physician (PCP)	6	14%	2	11%	1	25%	1	25%	2	20%
Primary Care Physician–HIV Specialty (PCP-HIV)	6	16%	5	26%	1	25%	0	0%	0	0%
Primary Care Assistant or Nurse Practitioner (PA, NP) or Registered Nurses (RN)^a^	7	16%	5	26%	1	25%	1	0%	0	0%

^a^Despite differences in background and training, these provider types are combined into a single category to protect identities of small numbers of providers in some provider categories

### Themes

We first describe themes within the domain *Current Practices*; we then present themes that emerged within the modified CFIR domains: *Characteristics of Statins, Characteristics of Providers, Inner Setting,* and *Patient Factors*.

### Domain 1: current practices

#### Theme 1: CVD risk assessment for PLWH is considered standard practice, but there are several impediments to regular assessment

When asked to describe current practices for assessing CVD risk among PLWH, most participants indicated that they regularly conduct lipid/cholesterol panels and assess for other risk factors, including diabetes, overweight, smoking and family history. This was true across all participant types and settings.

*I see it as a part of providing good primary care and always thinking about whether patients should be on a statin. I probably check cholesterol more than I should, telling people about it and exercise and healthy lifestyle and quitting smoking. I think it’s a very, very prominent part of my practice, just in general, controlling cardiovascular risk factors, counseling and thinking about optimizing prevention. (ID, FQHC)*

However, a handful of providers indicated that they are not always able to screen regularly for CVD risk among their PLWH patients due to several factors including having limited time, the clinic focus being more on treating HIV/AIDS than more general primary care, or lack of continuity between providers.

*I have to say that it is probably not the primary goal of the clinic just because we’re trying to do so many different things but as patients get older and they begin dealing with things like diabetes and hypertension it gets on [the] radar screen … (ID, CACC-Hospital)*

#### Theme 2: there is variation in and uncertainty around frequency of CVD risk assessment

Providers described variation in the frequency of lipid/cholesterol panels, with some indicating they do a CVD risk assessment with lab work for all new PLWH patients, some indicating they do this annually for PLWH patients, and others indicating they conduct the assessment at more frequent intervals such as every three or 6 months. Several participants indicated they are unsure of the recommended frequency for CVD risk assessment for PLWH or of the exact timing of their own assessments. A couple of participants suggested that assessment frequency would be greater and more consistent if driven by clinic protocols and performance measures. There was not a common CVD risk assessment protocol across PCPs within or across settings.

*Well, we try to do [CVD risk assessment], at the very minimum, it’s yearly. I like to do them a little bit more often, so six months or so. (PA/NP/RN, FQHC)*

*I get a complete set of labs, including the lipid panel, probably every three months. (PCP-HIV, CACC)*

#### Theme 3: Most providers use the atherosclerotic cardiovascular disease (ASCVD) risk calculator, but some are unsure of whether risk adjustments are needed for PLWH; practices vary within clinics and across providers

Several providers use the ASCVD risk calculator as a guideline for statin prescribing. Some providers said they use it all of the time “as is,” in a very straightforward way.

*I usually do a lipid panel. You know, obviously we know whether they’re smoking or not. Then I do a CVD risk calculation. If it’s 7.5% or higher, I offer them a statin. (PCP-HIV, FQHC)*

Other providers said they use the calculator on occasion “*just to plug in the numbers*” to facilitate discussing risk projections with patients.

*I like to go over all the results in the lipid panel, depending on their age, if they’re in that 40 to 70 or so range, then, if they are smoking or anything, I’ll talk about that, pull up the risk factors for it, and pull up the values right there, and just go in with them and say, “Hey, for the next ten years, this is what your risk would be,” and basically have that discussion with them, and that’s, you know, it’s going to, for the HIV patients, it’s another, I guess, I don’t remember all the tools they’re using for HIV, but I still plug in their other things, their smoking history, their age, their HDL values, their LDL values, and have that discussion with them. Now, I don’t do it on every single patient. It really depends on, you know, what I have on their history and then, what their lab values are. (PA/NP/RN, FQHC)*

Some providers adjust the risk threshold to account for increased risk for CVD from HIV; others expressed being uncertain about whether or how to adjust the calculator for HIV.

*I mean we do talk about cardiovascular risk appearing to be greater in people with HIV even treated and suppressed. And – but I still use that calculator as sort of like the best available tool and might consider their risk even higher depending on their comorbidities and their age, and then maybe the duration of HIV infection. (ID, FQHC)*

*After we obtain labs for example we generally do use, some people still use the Framingham, but other people use the ASCVD scores to try and calculate whether or not they would benefit from a statin but we also know that it does not factor in HIV into those the ASCVD, and Framingham scores. And sometimes we just base it on a personal judgement based on patients own risk factors, any family history etcetera, etcetera. (PCP, FQHC)*

*But it's probably more just about … we go off of like the lipid panel and ASCVD. Those are for the general population and not for HIV-specific populations. I'm not exactly sure for the coverage, and HIV-specific population guideline. (ID, CACC-Hospital)*

Similarly, some participants were unsure if substance use, such as methamphetamines, cocaine, and opioids, requires adjusting a patient’s risk score or statin treatment and noted lack of guidelines.

*I would say maybe the third [resource needed] would be lack of guidelines for how the guidelines are different for patients with HIV or patients who have used meth for years now. You would think that those presumably would be higher priority for statin use. There’s no real difference in the guideline right now I don’t think. (PCP-HIV, FQHC)*

A few providers said they never use the calculator. Some providers not using the calculator had an apologetic tone and wondered if they should be using it regularly.

*You know I honestly do not it use [the ASCVD risk calculator]. But I would say I should. But I'll be honest I do not use it. I think I use it once or twice in the last couple of years. So, no, to be honest, I don't. (PCP, CACC-Hospital)*

#### Theme 4: Most providers reported that they prescribe statins for their PLWH patients but there is variation in prescribing practices

When asked directly whether they regularly prescribe statins for their PLWH patients, most responded affirmatively. However, when and for whom this is done varied across providers and settings, with some focusing primarily on older patients and some, despite knowing the importance, missing the opportunity due to time and other constraints. (Reasons for variation and constrains are discussed in greater detail below, within CFIR categories.)

*I don't always feel super strongly that they go on a statin. So, I give them enough to allow them to help with their decision versus other patients, but I don't feel super strongly. You know, I rate them differently so that I ensure they get started on statin. So, I think it just depends on your patient population, because in general, my patient population being a little bit younger, definitely don’t want to take another medication. I don't feel super strongly about it … but in older people, I feel strongly, then I'll play it differently to work harder to get them on a statin, and usually, that can happen. (ID, CACC-Hospital)*

*I do [regularly prescribe statins]. I feel like if I don’t it’s because more of a time constraint that I missed it kind of thing rather than a lack of desire to do it. (PCP-HIV, FQHC)*

### Domain 2: characteristics of statins

#### Theme 1: participants believe that statins are effective for PLWH and easy to prescribe

Participants uniformly agreed that statins are effective in reducing CVD risk for PLWH.

*I mean, I think they are the best tool that we have. They do reduce most people’s cholesterol, and then they you know have an anti-inflammatory aspect as well, on top of the effects on the LDL directly. So, I think they’re pretty good. (ID, CACC-Hospital)*

Most participants believe statins are relatively easy to prescribe. Several participants described the overall process involving electronic ordering, communicating with the pharmacy, and billing as straightforward and easy to navigate. A few noted that prescribing statins is easier and more straightforward than prescribing other medications.

#### Theme 2: providers generally believe that stains are more effective than lifestyle changes alone

Although providers believe that statins are more effective than lifestyle changes alone, **s**everal providers typically have the patient try diet and exercise and smoking cessation before statins, as recommended by guidelines [31]. Some noted that many patients ask to try lifestyle modifications like diet and exercise prior to a statin; providers said they typically comply with patients’ preferences but are skeptical about the probability of success. They noted that most of their PLWH patients are low-income and do not have access to healthy food or places to exercise, limiting the chances of improving risk through diet and exercise alone.

*I mean we set sort of goals, like if their – if we can't modify their risk, like quantify it in terms of the cholesterol or stopping smoking in a certain timeframe, it's usually starts at it like six months. Then we will – you know, like as – like you have six months and then if we take it to where we need to be, then we'll start a statin. So, usually like a time frame on it. (ID, FQHC)*

*So I will first – and depending on how high their – how bad those [ASCVD score] numbers are, I will first talk to them about, like just healthier eating, exercise. I know that they may not have a lot of choices down here in these areas, just at the shelters. They don't have a lot of choice to food and what's served to them. And we can talk about whether or not they can make better choices with the limited options they have. So you don't have the second piece of cake … have one piece of cake or you drink water instead of soda. So we'll talk about things like that. For some of them where it's just not working, then I will go ahead and I will put them on medication and we'll still talk about, "You should still try to eat healthy. You should still try to exercise. This is not like, you can take a pill and do whatever you want." I've had some people actually think that. (PA/NP/RN, FQHC)*

#### Theme 2: many providers have concerns about drug-drug interactions (DDI) between statins and antiretroviral medications, but concerns typically do not limit statin prescribing

When asked if they had any concerns about statins for PLWH, concerns about DDI with HIV medications were mentioned by almost of half of all participants. Those who had these concerns didn’t indicate that they limited statin-prescribing. A few participants noted their belief that older HIV regimens have more DDI with statins.

*I think before all these newer HAART regimens there was always this question of can I prescribe a statin, is there drug-drug interactions, which statin is the best to use, which statin isn’t. And that’s probably more back in like 2015, or 2014 when it was little tougher. But kind of know with the newer single tablet regimen, it doesn’t seem to be that big of a deal. (PCP, CACC-Hospital)*

*If they’re on one [HIV regimen] that doesn’t really interact with the statins, then of course, it’s very easy. That’s really the wave of the future, if the patient’s on – have HIV patients on HIV regimens that don’t really interact with the liver system that metabolizes the statins, but there are going to be some people that are still on some of the older drugs that do interact. So, in those patients on the older regimens, where there’s the DDI, it can be a little bit more challenging to get them on, you know, perhaps more aggressive statins that do interact with that system. (ID, CACC-Hospital)*

Others noted concern about interactions with specific types of antiretroviral medications and noted that they choose the statin and dosage based on which HIV medications the patient is taking.

*Yeah, because we’re HIV [doctors], a lot of my patients are either on a booster PI or they’re on an integrase inhibitor. So generally we use Lipitor at a lower dose, but it’s either going to be Lipitor or Crestor. (PCP, Community Clinic)*

#### Theme 3: atorvastatin is the preferred statin for PLWH, but statin preferences and rationale for using them vary

Almost half of participants said they prefer prescribing atorvastatin for PLWH, citing effectiveness and fewer DDI with HIV medications. Pravastatin and rosuvastatin also were cited as preferred statins for PLWH.

*“Well, I think the ones here there’s atorvastatin, pravastatin, I think those are the ones that have less drug interactions than some of the other ones.” (ID, CACC-Hospital)*

Reasons for medication preferences include perceptions that some are more tolerable with older or any antiretroviral therapies (pravastatin); are more effective generally (atorvastatin, rosuvastatin), have the most evidence, greater availability and more insurance coverage (atorvastatin); are better for prescribing at lower doses (atorvastatin), and are better for people with “higher [ASCVD] numbers” (rosuvastatin). One participant noted that some PCPs prescribe simvastatin for PLWH, which he believed doesn’t work well for PLWH on antiretroviral therapy and noted difficulties when PWLH come to an HIV clinic with prescriptions from PCPs not treating PLWH.

### Domain 3: characteristics of providers

#### Theme 1: providers indicated knowledge gaps and a need for standard of care information

Participants had various concerns about statin prescribing for PLWH and indicated the desire for more information and data. In some cases, responses suggested a lack of knowledge in certain areas; in other cases, participants admitted their lack of knowledge and expressed the desire for more information about the standard of care for assessing CVD risk and prescribing statins to PLWH.

##### CVD risk threshold

Uncertainty related to CVD risk and statin indication adjustment for PLWH was the largest knowledge gap. When asked about risk assessment and statin prescribing practices for PLWH, about half of the participants expressed that they were unsure whether or how to adjust the ASCVD risk threshold for prescribing for PLWH; several also expressed the desire for a straightforward, simple algorithm for prescribing statins that takes into account risk for HIV.

*I mean, some are stronger than others. I know people who are living with HIV probably have a higher risk of cardiovascular disease, so I don’t know if they should be prescribed more. I think that hasn’t been really determined yet how we would preferentially give them more, how the calculation would be different. (PCP-HIV, FQHC)*

*Perhaps what I'd like to do is a very simple algorithm that could be used in when to start the medication .... I think I know most of it … So, I think kind of simple flow chart of describing statins, which statin is the preferred statin. And perhaps how to adjust it [FOR HIV], because that's something I don't even know. This is something that I should have also I'm guessing with, you know, meaning how much to go up by with that and so on and so on. (PCP, CACC-Hospital)*

##### CVD risk among and effect of statins on PLWH

Many providers expressed the desire for more information about statins and CVD for PLWH specifically. This seemed particularly important for ID specialists in primary care roles. As one clinic leader observed,

*If they’re going to make these [infectious disease] specialists take care of primary care, then there needs to be education on what statins are, how they work, when to take them, just general information and for the doctors to understand that just because they have HIV doesn’t mean that they’re not going to get other things. (CL)*

Several participants were unsure of how statins inhibit cardiovascular incidents or reduce risk for PLWH; a few specifically noted wanting more information on the quantity of risk mitigation statins provide to PLWH.

*I think it can potentially reduce it for 10 to 20 points, but I don't know in terms of how much in risk mitigation it is doing in for reducing cardiovascular disease risk. (ID, CACC-Hospital)*

##### Drug-drug interactions (DDI)

Several participants expressed needing more information about DDI, particularly interactions between statins and HIV antiretroviral medications.

*Yeah. I guess the one thing we didn't [discuss is the] interaction between the antiretroviral and their med, or I'm maybe mentioning other kinds of things, but I think at least right now with most of the antiretrovirals especially as we mainly use integrase-based regimen, the interaction with statins are quite known, and especially with using stuff like atorvastatin or rosuvastatin or I guess the new one, pitavastatin. So they're [interactions] not so much an issue anymore... So I guess also for that type of, what's kind of the best if a patient needs to be on a PI or Genvoya what should we do about the statin? I think that would be useful. (ID, CACC-Hospital)*

#### Theme 2: greater self-efficacy around communicating with PWLH about CVD and statins is needed to improve statin uptake

Several providers feel they don’t have adequate skills, time, or tools to counsel patients on CVD.

*I am going to do diet and exercise and off the bat, like at that encounter I encourage that, but I think sadly as a physician, me personally, and I think physicians are really well trained in prescribing diet and exercise, and we don't have a necessarily or I don't necessarily have those skills and time to adequately [provide] counseling.” (ID, CACC-Hospital)*

Nearly half of the participants were interested in more effective ways to communicate with patients about statins. Providers felt improving communication with patients would facilitate patient willingness to start on a statin.

*And so, I guess [what’s needed is] more of an easy solution to calculating a risk that help ensure decision making but then also I think kind of that ability to do shared decision-making, and kind of include statins in the package of therapies. (PCP, CACC-Hospital)*

Several providers were interested in how to better advise patients to address lifestyle changes. Participants described discussion of lifestyle changes as highly relevant to discussing statins.

*I mean, I think, you know, information on things that help people with lifestyle change, smoking cessation resources, you know, diet, exercise, especially for like, you know, low-income, you know, communities, where people struggle to pay for like, you know, food and other things like, you know, ways of managing and helping people improve, like, lifestyle and smoking cessation, I think would be really helpful, and I don’t really feel like that’s an area that I get anything from my community on stuff like that. (ID, FQHC)*

A few providers were unsure how to address or negotiate with patients who are hesitant about taking statins, and a few were interested in alternatives.

*Here's something … is there something else we can do other than statins? Like if somebody just clearly didn't want medication, are there alternatives, or something like that? (ID, CACC-Hospital)*

### Domain 4: inner setting

#### Theme 1: statin-prescribing is supported by clinic leadership and fits clinics’ mission and goals, but views about which providers “should” prescribe statins vary

Clinic leaders and providers support statin-prescribing for PLWH and agree that CVD risk management falls within the purview of their clinics and other clinics treating PLWH.

*Most of the HIV clinics are just like us – they’ve known for years about the risks of cardiovascular disease in HIV. So, almost all of the clinics that we know of, HIV doctors, they all give statins. It would be unusual if they weren’t giving them. (PCP, Community Clinic)*

However, some providers working with PLWH within primary care clinics do not view CVD risk assessment and management as their responsibility. This view was expressed more by ID specialists working with acutely ill PLWH. Some expressed deficits in training and self-efficacy as the reason for this while others expressed that their own specialty training and interests are not in the area of general primary care activities.

*To be honest, I think we think of ourselves as infectious disease specialists and not as primary care doctors. We're not as up on the evolution of diabetes management, hypertension, or cardiovascular risk tools, because they changed a lot. And we're busy keeping up in our sub-specialty. So I think there is some tension between the primary care role and the specialty role …I mean I became a specialist because I wanted to be a specialist and not because I wanted to manage people with diabetes.*

*(ID, CACC-Hospital)*

#### Theme 2: time and clinic and provider priorities are closely linked and affect whether CVD is addressed

Several participants described the amount of time they have available for patients as both a barrier and facilitator. Many reported that having extra time with patients facilitates statin prescribing, while others reported that not having enough time with PLWH patients poses a barrier. Some providers explained that there are too many things to go over with PLWH in their short appointment times to properly address CVD.

Whether or not providers have enough time with patients is closely tied with clinic and provider priorities. Clinics and providers focusing on acutely ill PLWH with unsuppressed viral load, AIDS, or other more pressing concerns for patients, noted that long-term CVD risk and statins are a lower priority. Care in some settings for acutely ill patients requires triaging that may preempt a focus on CVD and preclude statin initiation.

*I don't think I have concerns about prescribing [statins]. In terms of workflow, you know, sometimes I forget. It just depends on, you know, how many other things and what's more pressing. So it might be that it's important, but maybe I need to get them through ABC before I can get to D. Because the ABC are what's gonna hurt them first or that's what's causing them the most distress. (NP/PA/RN, FQHC)*

*Certainly if they were a well-controlled HIV patient, it fits into their workflow very well. I think, on the other hand, if you have a patient with whom is about to die from an infection, like a meningitis patient, we’re kind of more dealing with the more immediate life-threatening issues. Some of them were primary care issues, kind of are not as prioritized in that situation. We may not be addressing if their blood pressure is 150/85 and if their cholesterol is a little bit high, because, you know, any moment, they could die of meningitis, or something else, but in those patients who are well-controlled, and their infectious issues are not on the forefront, then I think it works very well within the workflow.(ID, CACC-Hospital)*

### Domain 5: patient factors

#### Theme 1: addressing patient psychosocial and housing issues can take precedence over CVD risk assessment and statins

Almost all providers noted that needing to address patients’ substance use, mental health and housing issues can interfere with addressing CVD and prescribing statins. Many providers discussed needing to prioritize these concerns during visits and that this interferes with the time needed to address and manage CVD.

*But we also have a small to moderate amounts of patients who are homeless or have a bit of psychosocial issues, and that can take priority over the visits. And so, the statin issue just gets pushed ‘til later. I would say that's probably the biggest issue. I mean, ideally, I think all of us would want to make sure everyone's top in mind as much as possible, but other things just come up (ID, CACC-Hospital)*

Providers also noted that psychosocial and housing issues all greatly affect the ability of PLWH to adhere to their HIV medications; many providers noted this challenge and said that if patients are not adhering to their HIV medications they likely cannot begin or adhere to a statin, or attend follow-up visits.

*Then we have other things to discuss. I think the other barrier is there are other things the patient is dealing with that are higher priority at the moment or it feels like they’re at a higher priority. Either they’re not taking their meds or they’re regularly using drugs or they’re homeless. Those things tend to be the things we focus on first to kind of stabilize the patient and get them to even take their HIV meds regularly. If they’re not taking their HIV meds regularly, me throwing on atorvastatin, probably they’re less likely to take it. If they are making an effort, I will put it on. (PCP-HIV, FQHC)*

Some providers also noted that low health literacy may interfere with willingness to take or ability to adhere to statins.

*The more important problem is to make sure they’ll take the statin, and that is just partly due to the kind of patients that I have. It’s a relatively poor environment up here. A lot of my patients are not well-educated. They don’t know much about medicines. A lot of them are homeless. They’re on drugs and so forth, and those kind of people are not adherent. I mean, they won’t even take their HIV meds. (PCP-HIV, CACC)*

Several providers noted an inherent paradox with these issues among PLWH—the patients who need statins the most are the ones who do not have the resources to adhere to them or to pursue lifestyle alternatives to lower their risk.

#### Theme 2: patients prefer trying to make lifestyle changes before taking a statin

More than half of the participants noted that their PLWH patients typically request trying to make lifestyle changes to lower their CVD risk, like smoking cessation, dietary changes, and exercise, prior to starting a statin. Lifestyle changes are an efficacious, guide-line recommended treatment to lower blood cholesterol [31]; however, many report that patients’ efforts typically aren’t successful. And, also noted earlier, many patients do not have access to healthy foods and places to exercise, further limiting chances of success. After unsuccessful efforts, patients may be more ready to initiate a medication.

*… I am just like look I know we’re doing due diligence in trying and attempting on these things [diet, exercise] on our own but it looks like we need a little help, I think in the meantime to be safe you know let’s do this. Let’s use a statin and use something else. And generally, after two or three visits of realizing they can’t do this on their own they do agree to start [the statin]. (PCP, FQHC)*

*I think they’re generally willing if we explain to them why it’s important. I think the younger patients are maybe a little bit more reluctant to start meds and they wanna give a trial of diet and exercise, some of them. And we would support that to see what happens. (ID, CACC-Hospital)*

#### Theme 3: providers believe most patients eventually are willing to take statins, particularly after shared decision-making

About half the providers across provider types and settings reported that patients typically are willing to do whatever their provider recommends, regardless of reservations. Only a few providers thought patients had had significant concerns about interactions with HIV medications and other side effects, a few thought patients might resist because they do not want to take another pill (in addition to their HIV and other medication), and a few noted occasional insurance barriers. But despite these concerns and other challenges PLWH may face, providers said their patients typically are willing to try a statin if they recommend it, particularly if the provider takes the time to lay out concerns about risk and engage in shared decision-making.

*I’ve had, I’d say, a very good [uptake]. I’ve had a handful of patients who they’d rather not take another pill, or they want to work on their diet. I can then say I’ll cut down on, you know, whatever they need to. But I would say 90% of patients are accepting of it, once you explain, you know, the rationale, and why it’s important. (PCP-HIV, FQHC)*

*You know it’s shared decision-making, and …it is just kind of laying out the reasons for why the medication is indicated try to educate the patient on their risk, to make sure they have a good understanding of it, and then just kind of leave the decision up to them.(PCP, CACC-Hospital)*

One administrator noted the importance of the provider’s relationship with patients in facilitating treatment:

*I think in general the patients here love the providers, and if a provider told them to jump into cold water every day because it was going to make them better, they would probably do it. (CL).*

## Discussion

Using an implementation science framework—the CFIR—this study qualitatively assessed and categorized themes potentially related to low statin uptake for PLWH from interviews conducted with clinic leadership and providers in community health clinics across Los Angeles County, California. While ASCVD risk assessment and statin prescribing typically fall within clinics’ mission and goals and are considered standard primary care practice by almost all participants, statin uptake may be impeded by multiple factors across CFIR categories. Barriers include provider knowledge and self-efficacy gaps (CFIR Characteristics of Providers and Characteristics of Statins), clinic and provider workflow and priorities (CFIR Inner Setting), and patient-level factors (CFIR Outer Setting).

We found that while most providers do report conducting CVD risk assessment and prescribing statins regularly for their PLWH patients, practices vary widely across providers and clinic types, and knowledge and self-efficacy gaps may impede these practices. For example, many providers desire more data about the relationship between HIV and CVD and more information about whether and how to adjust the ASCVD risk threshold for HIV and for other comorbidities common among some PLWH, like substance use. Moreover, although participants strongly believe statins are effective and safe for PLWH, there is great variation in perceptions of which statins are best for PLWH with respect to DDI with antiretroviral therapy. Self-efficacy issues may also impede prescribing, with some providers feeling they do not have adequate skills to discuss risk and shared decision-making. This is consistent with prior studies of providers who care for PLWH, in the context of their evolving roles as their patients experience increasing life expectancies and comorbidities [1]. These providers tend to report lower comfort levels in primary care services compared to colleagues who treat the general population [32–35].At the same time, shared decision-making and clear discussion of risk is thought to be one of the strongest facilitators to statin uptake. Thus, providing more targeted distribution of standard practice information on CVD risk among PLWH as well as integrating protocols for risk assessment and statin-prescribing and shared decision-making into clinical practice could facilitate statin uptake.

Clinic workflow and clinic and provider priorities also may impede statin prescribing; this seems to vary by type or emphasis of clinic. For example, for clinics and providers that focus primarily on acutely ill PLWH, CVD assessment and stains are less of a priority, either because of lack of time to address it or because the other problems take precedence over statins. Also, some noted that CVD risk assessment is not a performance measure at their clinic, which for some may be an important driver of regular practices. Some providers—ID specialists in particular—do not believe that CVD risk assessment or statin prescribing and management falls within their purview, even within primary care settings, and prefer not to do it because of lack of training or expertise. This is consistent with prior studies reporting that providers who care for PLWH who do not typically provide primary care have more reservations about providing primary care services [26, 32]. Nevertheless, most providers in this study do feel it is both within their purview and an important priority for PLWH, as long as time allows. The adoption of guideline-based statin prescription for the general population also has been a challenge [34, 35], and the literature offers several interventions that can be implemented to accommodate patient and provider preferences and clinic resources. These interventions also may apply to improving uptake of statins for PLWH. While clinic visit time often is an impediment to uptake of many practices in primary care and difficult to change [36, 37], having one additional visit with the PCP annually (e.g. 2 visits vs 3 visits) was associated with higher odds of evidence-based statin use among general primary care patients, suggesting that having the additional visit allowed the patient to discuss statin therapy with the PCP and lead to more statin prescriptions [38]. Other facilitators could include standard operating procedures, EMR tools, and performance measures and feedback around CVD risk assessment and statins [39–41]. For example, the use of a multi-component intervention targeting providers, including decision support, brief education, performance measures, and feedback, was shown to increase the adoption of statin prescription in primary care settings for the general population [42]. Another strategy may be team-based models, such as engaging nurses in initiating CVD risk assessment and providing recommendations [43], or bringing primary care providers into the clinic whose role is to manage complex non-AIDS comorbidities and provide primary care if preferred by the HIV provider [36].

Patient-level factors such as psychosocial and housing issues and low health literacy were noted by most providers as impediments to both statin uptake and adherence. For some providers, a focus on these issues during visits precludes addressing CVD during regular clinic visits. Providers also perceive that these issues affect adherence to HIV and other medications, and they are reluctant to prescribe another medication that may not be taken. Patients also may request to try making diet and exercise changes prior to taking a statin, but providers note that due to many of their PLWH patients living in low-resource communities, lifestyle changes typically are not successful in lowering cholesterol levels. While mental health, substance use, and housing, among other issues, can impact uptake of and adherence to HIV treatment among PLWH [44, 45] and may also impact statin uptake, providers noted that care coordinators, medical case managers, pharmacists and nutritionists who help PLWH with their HIV medications also can facilitate statin uptake and adherence. Given the range of possible impediments to prescribing statins for PLWH, integrating specific guidelines and protocols around statin-prescribing into clinic workflows may be most useful for facilitating prescribing decisions.

Overall, perceptions of statin effectiveness and safety are positive and willingness to prescribe them to PLWH are high among this diverse sample of primary care providers and clinic leaders, despite some notable impediments. Of course, these qualitative data do not include prescribing rates, so it is difficult to be sure if providers’ views of their own prescribing practices match actual practices. In future research we aim to assess this relationship, as well as patient perspectives of and willingness to take statins. Additionally, the parent study – INSPIRE—will assess the impact on prescribing rates of receiving an intervention combining CVD risk and statin education as well as feedback about prescribing practices.

### Limitations

Our study has some limitations. First, our sample was not randomly selected and is not representative of clinic leadership or providers who care for PLWH in all community health settings. Clinic leaders and providers in clinics that agreed to participate in the larger INSPIRE study all were invited to participate. Nevertheless, only two providers declined the invitation. Further, the participants are diverse in training and credentials and the eight clinics span across Los Angeles County, the most diverse and populous in the country, thus increasing the potential for generalizability. Second, providers were aware that the interviews were being conducted as part of a larger study aimed at increasing statin uptake through education and feedback, thus responses may have been biased to reflect more positive views and practices. That said, barriers and deficits were openly discussed, and providers were aware they would not be identified. Additionally, interviews were conducted over a year-long period, thus providers that were interviewed later may have begun to increase their knowledge and change their practices as they learned about the study. Finally, the manuscript is missing the patient perspective; a forthcoming manuscript will report on finding from focus groups with PLWH. Limitations notwithstanding, the study fills a gap in the implementation science, HIV, and CVD literature about barriers to statin uptake for PLWH and highlights important knowledge and information gaps.

## Conclusions

Providers desire more data and standard practice guidance on prescribing statins for PLWH, including estimates of the effect of HIV on CVD, how to adjust the CVD risk threshold to account for HIV, which statins are best for people on antiretroviral therapy, and on shared decision-making around prescribing statins to PLWH. While CVD risk assessment and statin prescribing fits within the mission and workflow of primary care, clinics may need to emphasize CVD risk assessment and statins as priorities in order to improve uptake. Where there is a poor fit between provider expertise and training and statin-prescribing, some clinics may wish to designate certain providers to assess CVD risk and prescribe statins. In light of the evidence suggesting the effectiveness of statins primary prevention of CVD in PLWH, it is imperative that primary care providers integrate CVD risk assessment and statin prescribing into their routine practices when caring for PLWH.

## Data Availability

The datasets used and/or analyzed during the current study are available from the corresponding author on reasonable request.

## Abbreviations

CVD: Cardiovascular Disease
PLWH: People Living with HIV
FQHC: Federally Qualified Health Center
CACC: County Ambulatory Care Clinic
CFIR: Consolidated Framework for Implementation Research
CL: Clinic Leadership
ID: Infectious Disease Specialist
PCP: Primary Care Physician
PCP-HIV: Primary Care Physician – HIV Specialty
PA: Physician Assistant
NP: Nurse Practitioner
RN: Registered Nurse
ASCVD: Atherosclerotic cardiovascular disease
